# Altered ocular parameters from circadian clock gene disruptions

**DOI:** 10.1371/journal.pone.0217111

**Published:** 2019-06-18

**Authors:** Richard A. Stone, Alice M. McGlinn, Ranjay Chakraborty, Duk Cheon Lee, Victoria Yang, Ayman Elmasri, Erica Landis, James Shaffer, P. Michael Iuvone, Xiangzhong Zheng, Amita Sehgal, Machelle T. Pardue

**Affiliations:** 1 Department of Ophthalmology, University of Pennsylvania Perelman School of Medicine, Philadelphia, Pennsylvania, United States of America; 2 Department of Ophthalmology, Emory University School of Medicine, Atlanta, Georgia, United States of America; 3 Center for Visual and Neurocognitive Rehabilitation, Atlanta VA Medical Center, Decatur, Georgia, United States of America; 4 Department of Biomedical Engineering, Georgia Institute of Technology, Atlanta, Georgia, United States of America; 5 Department of Pharmacology, Emory University School of Medicine, Atlanta, Georgia, United States of America; 6 Department of Neuroscience, University of Pennsylvania Perelman School of Medicine Philadelphia, Pennsylvania, United States of America; McGill University, CANADA

## Abstract

The pathophysiology of refractive errors is poorly understood. Myopia (nearsightedness) in particular both blurs vision and predisposes the eye to many blinding diseases during adulthood. Based on past findings of diurnal variations in the dimensions of the eyes of humans and other vertebrates, altered diurnal rhythms of these ocular dimensions with experimentally induced myopia, and evolving evidence that ambient light exposures influence refractive development, we assessed whether disturbances in circadian signals might alter the refractive development of the eye. In mice, retinal-specific knockout of the clock gene *Bmal1* induces myopia and elongates the vitreous chamber, the optical compartment separating the lens and the retina. These alterations simulate common ocular findings in clinical myopia. In *Drosophila melanogaster*, knockouts of the clock genes *cycle* or *period* lengthen the pseudocone, the optical component of the ommatidium that separates the facet lens from the photoreceptors. Disrupting circadian signaling thus alters optical development of the eye in widely separated species. We propose that mechanisms of myopia include circadian dysregulation, a frequent occurrence in modern societies where myopia also is both highly prevalent and increasing at alarming rates. Addressing circadian dysregulation may improve understanding of the pathogenesis of refractive errors and introduce novel therapeutic approaches to ameliorate myopia development in children.

## Introduction

The prevalence of myopia (nearsightedness) is high and continues to increase to alarming levels, particularly in developed countries [1]. If current trends continue, nearly half of the world’s population may be myopic by 2050 [2]. In normal eyes, an active homeostatic process coordinates the length of the developing vertebrate eye with the image formed by the combined optical powers of the cornea and lens to maintain visual images at the retinal photoreceptors. The control of refractive development is largely localized to the eye itself, with visual feedback and guidance mechanisms involving intrinsic retinal signaling [3]. Failure of this homeostatic process results in refractive errors. Myopia is the most common refractive disorder, where eye lengthening during childhood is inadequately compensated by the optical powers of the cornea and lens and distant images focus anterior to the retinal photoreceptors. Besides visual disability from blurred images, myopia comprises a major public health problem because the enlarged myopic eye is predisposed to many blinding diseases in adulthood, including numerous retinal pathologies, glaucoma and some forms of cataract. Because of these diseases, myopia is a leading risk factor for blindness [4–6], and none of these associated diseases are expected to be reduced by any of the available optical or surgical approaches to correct the image defocus. Despite evidence for a genetic contribution to myopia [7,8], the rapid increase in its prevalence in many regions of the world [1,2,9] implies non-genetic environmental and/or behavioral etiologies. Surprisingly, however, modern clinical research is failing to substantiate meaningful contributions from many presumed behavioral causes of myopia, such as altered accommodation or intensive use of the eyes for near tasks [7].

Among many hypotheses for the etiology of myopia, the potential role of inadequate lighting or insufficient exposure to the outdoors was first proposed in the nineteenth century [10–12], and this idea is again generating much laboratory and clinical interest. Intense laboratory lighting lessens experimental myopia in several vertebrate species [13]. Some feature(s) of outdoor exposure seems to partially protect against myopia in children [14–18]. Whether the anti-myopia attribute of outdoor exposures relates to lighting intensity, as currently hypothesized, or instead relates to some other property of being outdoors remains to be established.

Potentially related to lighting exposures, much evidence also links daily rhythms in the eye to refractive development. The dimensions of the eye undergo diurnal fluctuations in laboratory animals [19] and in humans [20–22]. These fluctuating dimensions include axial length, vitreous chamber depth and choroidal thickness, all of which may be pertinent to refractive development. Experimental myopia in animals [19] or imposed optical defocus in humans [23, 24] alter these rhythms in ocular dimensions.

Besides diurnal rhythms in ocular dimensions, rhythms in retinal signaling and molecular biology are linked to refractive development. Retinal dopamine signaling entrains many diurnal intra-retinal processes to the light:dark cycle, even the overall state of retinal light:dark adaptation [25,26]. The turnover of retinal dopamine affects such retinal rhythms, but it also influences refractive development [22,27,28]. Further supporting a role for daily rhythms in refractive development, the expression in combined retina/RPE (retinal pigment epithelium) of clock and/or circadian rhythm-related genes is altered in two experimental myopia models in chick [22,29,30] and in the RPE of a myopia model in tree shrew [31]. While controversial when first proposed, myopia has been associated with early childhood exposure to ambient lighting at night [22,32], a parameter potentially disrupting the circadian clock.

Taken together, these reports suggest that the control of ocular refraction may be linked to circadian rhythms, which are sensitive to, and could account for, the effects of outdoor and light exposures on refractive development. Accordingly, we have hypothesized that the endogenous retinal clock may connect both visual input and retinal *Zeitgebers*, such as light, to ocular rhythms and refractive development and that study of circadian mechanisms may lead to a biological explanation for the apparent anti-myopia effects of daytime light exposures [22,33].

To test the role of circadian rhythms, we investigated the morphological and refractive effects of clock gene disruption on optical development, using two widely separated species commonly used for circadian studies: mice, a vertebrate increasingly used to address the mechanisms of myopia; and *Drosophila melanogaster*, an organism frequently used to dissect the molecular genetics and physiology of the visual system but not yet applied to study mechanisms of refractive disorders. We find that mice with a retinal specific knockout of *Bmal1*, a non-redundant component of the circadian clock [34], have myopia with a lengthened vitreous chamber, the optical compartment separating the lens and retina. These changes simulate key findings commonly found in human myopia. Knockout of either the *cycle* or *period* gene in the circadian clock of *Drosophila* elongates the ommatidia pseudocones, an optical component of the fly eye that separates the facet lens and photoreceptors and insures focusing of images on photoreceptors. Based on these results, we propose that the circadian clock influences the pathways that control ocular development and that expanding clinical research from light exposures *per se* to the broader question of the ocular effects of circadian rhythm disruptions in modern societies may well provide a useful approach to understand and ultimately to ameliorate myopia.

## Materials and methods

### Ethics statement

The mouse protocols were approved by Institutional Animal Care and Use Committees (IACUC) of the Atlanta Veterans Health Care System, Emory University (Protocol V017-17), and the University of Pennsylvania (Protocol 805415); and they conformed with the NIH Guide for the Care and Use of Laboratory Animals and the ARVO (Association for Research in Vision and Ophthalmology) Statement on the Use of Animals in Ophthalmic and Vision Research.

### *Bmal1* knockout and control mice

All mice were maintained under typical laboratory conditions in standard plastic mouse cages (18cm wide X 29cm long X 13cm high) with *ad libitum* food and water and with unrestricted visual input on a 12hr light:12hr dark cycle with illumination from Octron@800 Ecologic fluorescent bulbs (4100K; Sylvania, Danvers, MA), providing approximately 70 lux at mouse eye level on position in cage.

*Bmal1*^*fl/fl*^ mice [35], in which exon 8 of the *Bmal1* (*Arntl*) gene was flanked by two loxP sites, were obtained from The Jackson Laboratory [Bar Harbor, ME; (B6.129S4(Cg)-*Arntl*^*tm1Weit*^/J)]. These mice were backcrossed with C57BL/6J mice at least 6 generations at the Jackson Labs. The conditional *Bmal1* mice were bred with mice expressing a single copy of Cre-recombinase driven by the *Chx-10* promoter [Stock Tg(Chx10-EGFP/cre,-ALPP)2Clc/J; The Jackson Laboratory], which is expressed in retinal progenitor cells during development and in inner nuclear layer cells in adults [36, 37]; the *Chx10-Cre* mice were backcrossed with C57BL/6 mice for 4 generations. Thus, the *Bmal1* gene was disrupted from retinal cells in mice expressing both *Bmal1*^*fl/fl*^ and *Chx10-Cre*, producing the retinal-specific *Bmal1* knock-out (i.e., *rBmal1* KO) mice. Two control genotypes were studied: littermates of the *rBmal1* KO mice without Cre and expressing only *Bmal1*^*fl/fl*^ (i.e., *Bmal1*^*fl/fl*^ mice), and the *Chx10*^*cre*^ mice with wildtype *Bmal1* alleles (i.e., *Chx10*^*cre*^ mice) to which the *Bmal1*^*fl/fl*^ were bred. Mice were genotyped by polymerase chain reaction in house and by Transnetyx, Inc. (Cordova, TN).

### Measurements of mouse eyes

We examined the refractive development of *rBmal1* KO and the control mice starting at post-natal day 28 (p28) until post-natal day 70 (p70) (n = 7 *rBmal1* KO; n = 10 *rBmal1*^*f//fl*^ controls; n = 13 *Chx10*^*cre*^ controls). Starting at baseline (p28) and at every two weeks, we measured refractive error, corneal curvature, and ocular axial parameters using photoretinoscopy, keratometry and optical coherence tomography, respectively, as previously described [38] and summarized in the S1 File. Examinations were all conducted within a four-hour window in the light cycle. At the end of the study, mice were euthanized using cervical dislocation by personnel trained to be proficient in this technique, and the retinas were collected for analysis independent of the current study.

### Mutant and control *Drosophila*

We studied genetic null mutants of two circadian clock genes on the wild-type Canton-S background: *cyc*^*01*^ (null mutation of cycle, a positive regulator in the molecular clock); and *per*^*01*^ (null mutation of period, a negative regulator) [39–41]. Each *Drosophila* genotype was maintained under a 12hr light:12hr dark cycle (cool white fluorescent light, 500 lux) at 25° C. Female flies were collected on day 5 or day 20 during the light phase, when the heads were fixed, embedded, sectioned, stained for visualization, and photographed. From the photomicrographs, one eye from 10 flies in each cohort was chosen for analysis using central tissue sections through ommatidia visualized in approximate full length. Two cohorts of 5 day-old wild type and *cyc*^*01*^ *Drosophila* were included. The anterior, central and posterior regions of each horizontal section of each eye were analyzed separately using between 6 and 10 ommatidia/fly for each region.

Using Fiji software [42], individual facet lenses were outlined and modeled as an ellipse. The diameters of the facet lenses (defined as the dimension perpendicular to the optic axis) and their thicknesses (defined as the dimension parallel to the optic axis) were estimated using the major and minor axes of the best-fit ellipses, respectively. The radius of curvature at the anterior vertex of the best-fit ellipse was estimated [43] using the relation: curvature = [(major axis/2)^2^]÷(minor axis/2). From the same ommatidia, the lengths of the pseudocones were estimated from a line extending from the posterior-most edge of the facet lens to the deepest location of the pseudocone. Further detail is provided in the S1 File.

### Statistical analysis

Unless otherwise specified, data are reported as mean ± S.E.M. For the mice, the mean values of both eyes for each parameter, for each mouse and at each time were used for analysis. Because of the challenges of measuring small mouse eyes, some parameters were successfully measured on only one eye of a specific mouse on a specific day; in these instances, the available monocular parameters were used in the analysis. Comparisons between the genotypes were performed by two-way repeated-measures analysis of variance (ANOVA) with Holm-Sidak post-hoc comparisons when a significant genotype X time interaction was identified (SigmaStat, San Jose, CA).

Using SAS (version 9.4; SAS Institute, Cary, NC) for the *Drosophila* data, multiple measurements of each parameter from the same region within an eye were averaged to create one summary data point for each parameter for each region of each eye. Data were summarized by parameter, age and genetic group using means and empirical standard errors. Because of the challenges of measuring sectioned fly eyes, some cohorts included only 9 flies. To account for the replicate measurements (across three regions) within an eye, we conducted overall and post-hoc statistical comparisons using linear contrasts of least squares means derived from mixed models with a random effect for fly and fixed effects for the genotype group, region, and interaction of genotype group and region, considering p<0.05 as statistically significant. None of the interactions were statistically significant.

## Results

### Loss of the retinal circadian clock produces myopia in a mouse model

To assess the role of the retinal clock in refractive development of the mammalian eye, we used the Cre-lox system to ablate the *Bmal1* circadian clock gene in the retina. Three cohorts of mice were studied: retinal-specific *Bmal1* knockout mice (*Chx10*^*cre*^−*Bmal1*^*fl/fl*^; hereafter referred to as “*rBmal1* KO” mice) and two control groups: 1) *Bmal1*^*fl/fl*^ mice (i.e., littermates of the *rBmal1* KO mice expressing only *Bmal1*^*fl/fl*^); and 2) *Chx10*^*cre*^ mice (carrying the eye-specific Chx10-CRE transgene, and to which the *Bmal1*^*fl/fl*^ mice were bred). Ocular refraction depends on complex interactions of the focusing properties of the cornea and lens and the anatomical dimensions of the components of the eye. The *rBmal1* KO mice differed from each of the control groups. The ocular development of the *rBmal1* KO mice is compared primarily to the *Bmal1*^*fl/fl*^ mice because these two genotypes are littermates and because the refractive development of *Bmal1*^*fl/fl*^ mice more closely follows those of unaltered C57BL/6J mice [44], the suggested control for the mouse with the floxed *rBmal1* allele used here [45]. The comparison of *rBmal1* KO to *Bmal1*^*fl/fl*^ KO mice is described here, while the S2 File addresses eye development of *Chx10*^*cre*^ mice.

**Fig 1:**
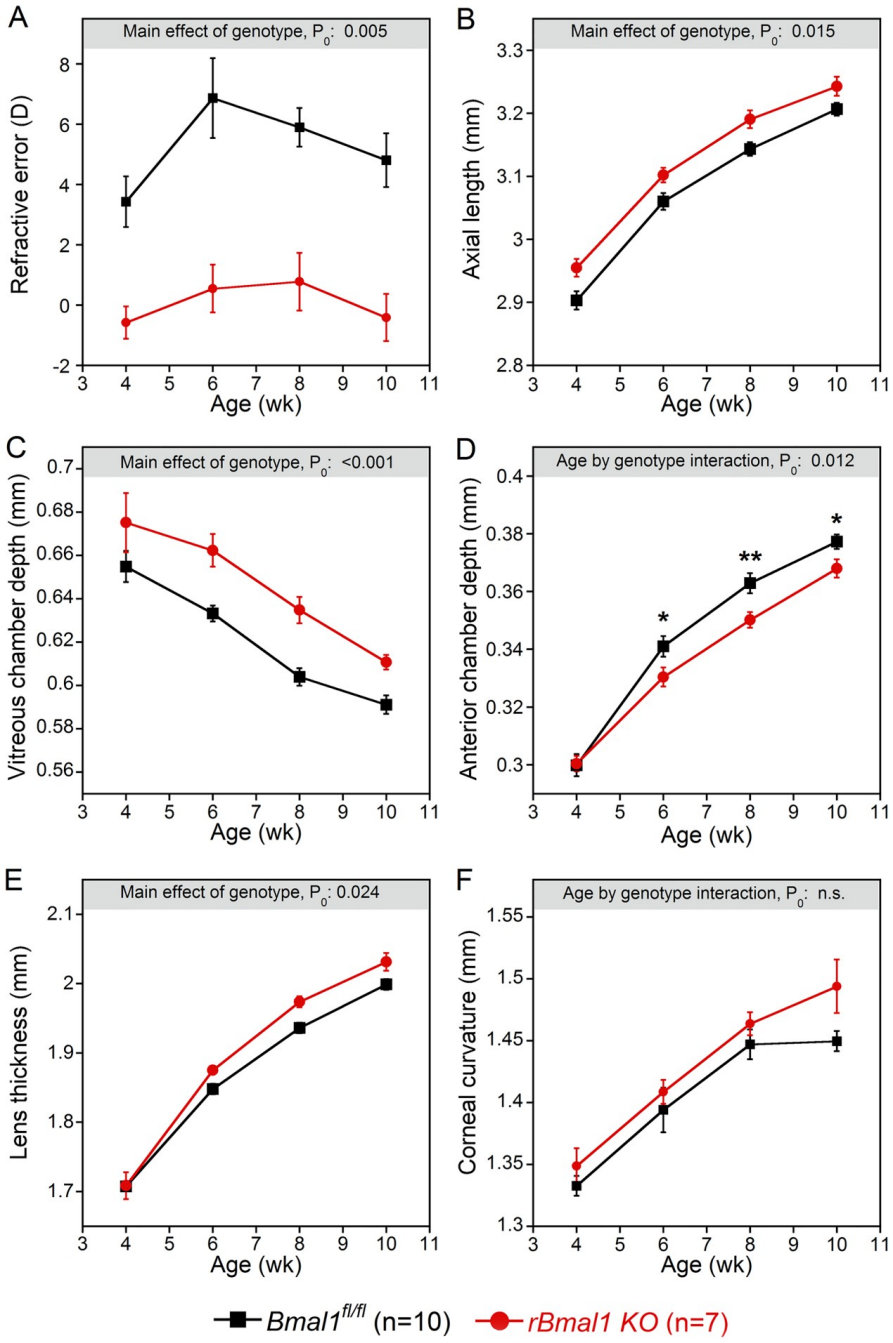
Ocular development in mice with retina-specific knockout of the *Bmal1* gene (*rBmal1* KO mice) compared to their littermate controls (*Bmal1*^*fl/fl*^ mice). A. The refractions of *rBmal1* KO mice are shifted negatively, i.e., significantly more myopic, compared to the *Bmal1*^*fl/fl*^ controls across all ages tested (P_0_ = 0.005). B. The absence of *rBmal1* results in an elongated axial length of the eye (P_0_ = 0.015). C. While the vitreous cavity depths decrease during the experimental period in both genotypes, the vitreous cavity depths of the *rBmal1* KO mice are consistently longer than those of the *Bmal1*^*fl/fl*^ controls (P_0_<0.001). D. The anterior chambers of the *rBmal1* KO mice deepened less than those of the *Bmal1*^*fl/fl*^ controls at 6 weeks and remained shallower at subsequent times. (P_0_ = 0.009). E. A greater lens thickness of *rBmal1* KO than *Bmal1*^*fl/fl*^ controls developed by 6 weeks of age and increased with age (P_0_ = 0.024). F. Corneal curvatures are equivalent for *rBmal1* KO mice compared to *Bmal1*^*fl/fl*^ control mice (P_0_, n.s.). P_0_ specifies ANOVA assessments for either inter-genotype comparisons or the interaction of genotype by age; post-hoc comparisons for the genotype by age interactions: *p<0.05, **p<0.01, ***p<0.001. Data appear in S1, S2 and S3 Tables.

**Fig 2:**
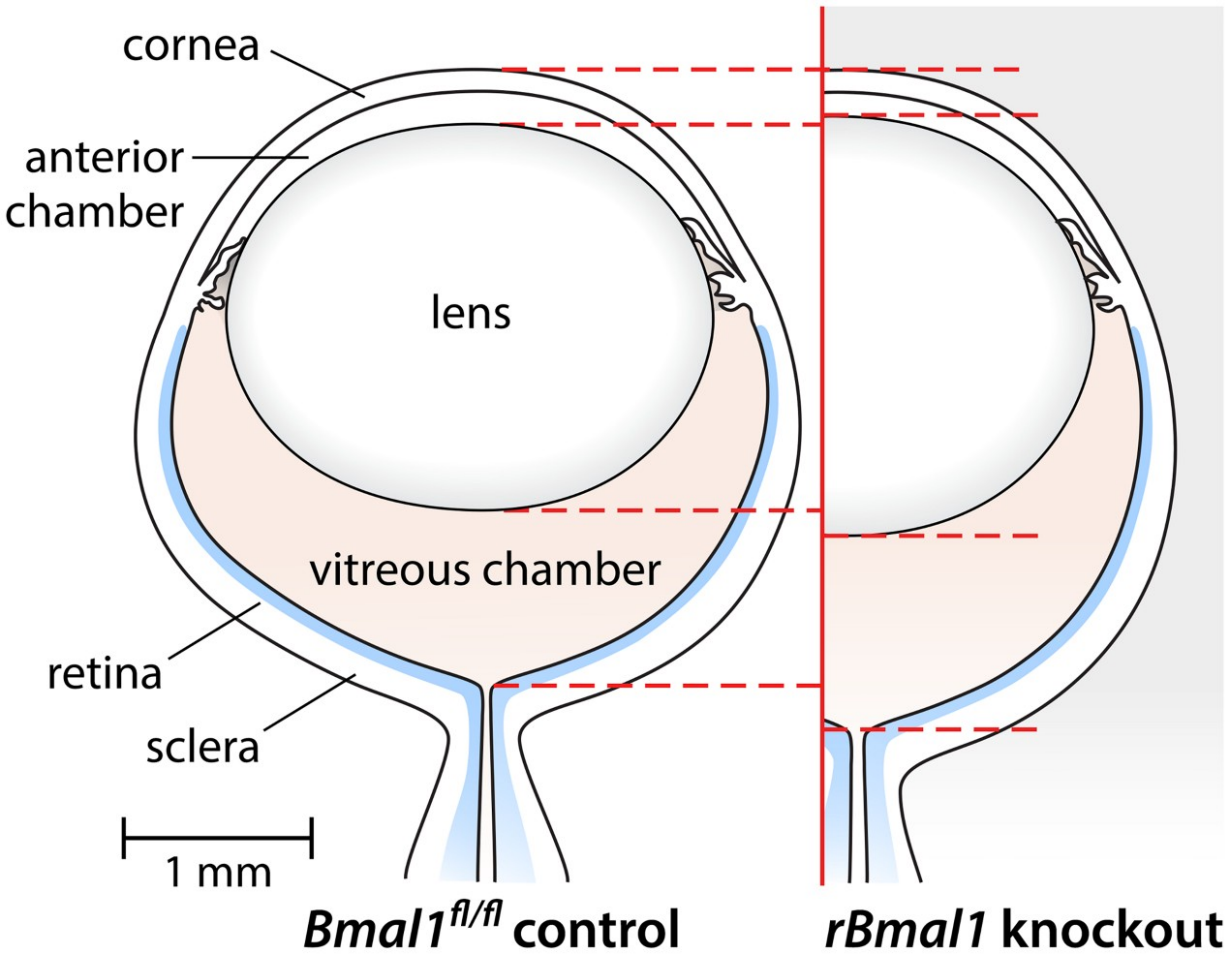
Schematic illustration of principal anatomical alterations in the mouse eye, comparing the *Bmal1*^*fl/fl*^ control mice to mice with retinal-specific knockout of the Bmal1 gene (*rBmal1* KO). Compared to their littermate *Bmal1*^*fl/fl*^ controls, the *rBmal1* KO mice develop shallower anterior chambers, thicker lenses, and longer vitreous chambers, resulting in overall longer axial lengths. Data appear in Fig 1 and in S1, S2 and S3 Tables.

Refractions and most ocular parameters determining refraction differed between the *rBmal1* KO and control *Bmal1*^*fl/fl*^ mice (Figs 1 and 2, with detailed data in S1, S2 and S3 Tables). The refractions of *rBmal1* KO mice were more myopic than the control *Bmal1*^*fl/fl*^ mice at all ages (Fig 1A, p<0.001), by an average of 4.01±0.67 diopters throughout the study. Consistent with their myopic refractions, the eyes of *rBmal1* KO mice had significantly longer axial lengths than those of the control *Bmal1*^*fl/fl*^ mice (Fig 1B, p = 0.015), averaging 0.044±0.005 mm longer over the ages tested. This increase in axial length was accompanied by significantly greater vitreous chamber depths in *rBmal1* KO compared to the control *Bmal1*^*fl/fl*^ mice (Fig 1C, p<0.001), that averaged 0.025±0.004 mm longer during the study. The corneal radii of curvature (Fig 1F, p>0.1) were comparable in *Bmal1* KO mice compared to those in these control mice.

Both the anterior chamber depths and lens thicknesses were similar between these two genotypes at 4 weeks of age, but each evolved differently over time. The anterior chamber depths of *rBmal1* KO mice deepened more slowly than those of control *Bmal1*^*fl/fl*^ mice. The lenses of *rBmal1* KO mice became significantly thicker over time than those of control *Bmal1*^*fl/fl*^ mice (S2 Table, Fig 1E, p = 0.024). The absence of *rBmal1* expression also reduced the total retinal thickness compared to control mice (S2 Table, p<0.003). While increasing over time in both genotypes (S2 Table, p = 0.01), corneal thicknesses were similar between the mutant and control mice.

### Loss of the circadian clock in *Drosophila* alters ommatidium development

**Fig 3:**
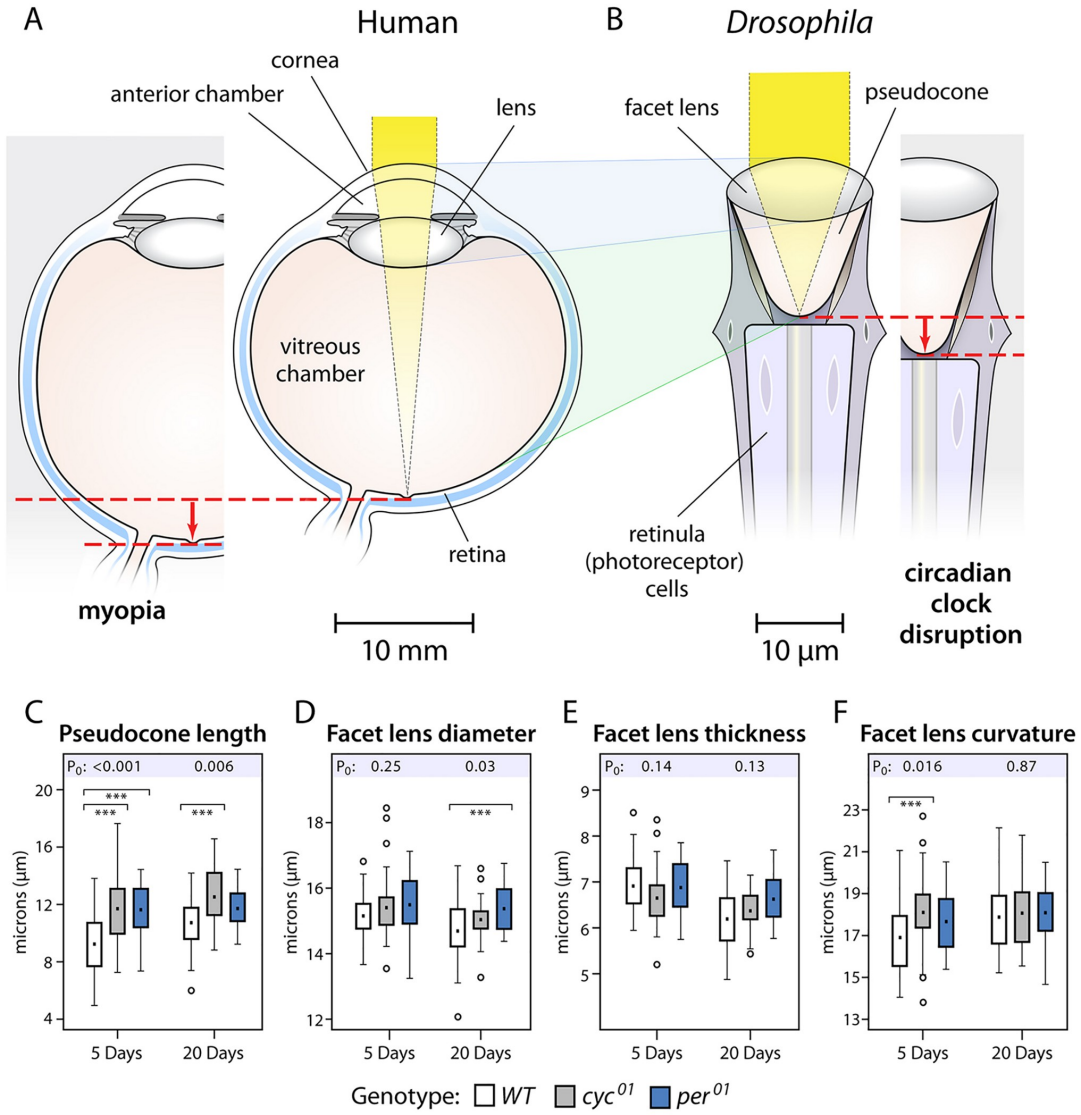
Circadian clock disruption and the morphology of the anterior part of *Drosophila* ommatidia. **A & B.** Schematic drawings comparing the human eye and clinical myopia to the *Drosophila* ommatidium in flies without and with a clock gene knockout. The cornea-(anterior chamber)-lens complex of the human eye (**A**) corresponds to the facet lens of the *Drosophila* ommatidium (**B**); the human vitreous chamber, to the *Drosophila* pseudocone. These optical components focus incoming light on the human retina or on *Drosophila* retinula cells. Human myopia most commonly results from vitreous chamber elongation (**A, left**). Clock gene mutations result in lengthened pseudocones (**B, right**), paralleling the main abnormality in human myopia. Dashed red lines and arrows facilitate comparison of the normal to altered structures in both species. Magnification bars illustrate the marked scale differences between the imaging structures of these species. **C.** At 5 days of age, the pseudocone length is greater in flies with null mutations of the clock genes *cycle* or *period* (*cyc*^*01*^ or *per*^*01*^), relative to control wildtype (*WT*) flies. At 20 days, the pseudocone lengthening remained statistically significant for the *cyc*^*01*^ flies but became a trend for the *per*^*01*^ flies (p = 0.07). **D.** The facet lens diameters are not affected at 5 days; at 20 days, the facet lens in *per*^*01*^ but not *cyc*^*01*^ flies was modestly wider than in *WT* flies. **E.** Facet lens thicknesses were unaffected at either age. **F.** The radius of curvature of the facet lens was longer in mutants compared to *WT* flies at 5 days; by post hoc testing, this lengthening reached statistical significance for the *cyc*^*01*^ flies. By 20 days, the curvature effect was no longer evident. For each cohort, the numbers of flies and ommatidium regions measured appear in S4 and S5 Tables. Box plots: solid squares, means; rectangular area, 25–75 percentiles; fences, 1.5X (interquartile range); open circles, outliers. P_0_, p-values of the overall statistical effect for each group. Bars, within-age statistically significant comparisons by post hoc testing: ***p≤0.001. Data appear in S4 and S5 Tables.

The basic molecular framework underlying circadian rhythms as well as physiological outputs of the clock are conserved from *Drosophila* to mammals [41]. To determine whether the *Drosophila* clock affects refractive development of its eye, we examined the ommatidia of clock mutants in *Drosophila*. A *Drosophila* eye contains some 750 ommatidia, with the anterior part of each ommatidium containing two major components [46]. The facet lens (also termed corneal lens) is a fixed-focus lens with approximately 5X10^4^ diopters of power; beneath the facet lens is the fluid-filled pseudocone (also termed crystalline cone). These optical elements concentrate incident light at the distal end of the rhabdomeres, specialized membrane modifications of the retinula (photoreceptor) cells. Optically, the facet lens corresponds to the variable-focus cornea-(anterior chamber)-lens complex in vertebrate eyes; the pseudocone is analogous to the vertebrate vitreous chamber (Fig 3A and 3B).

**Fig 4:**
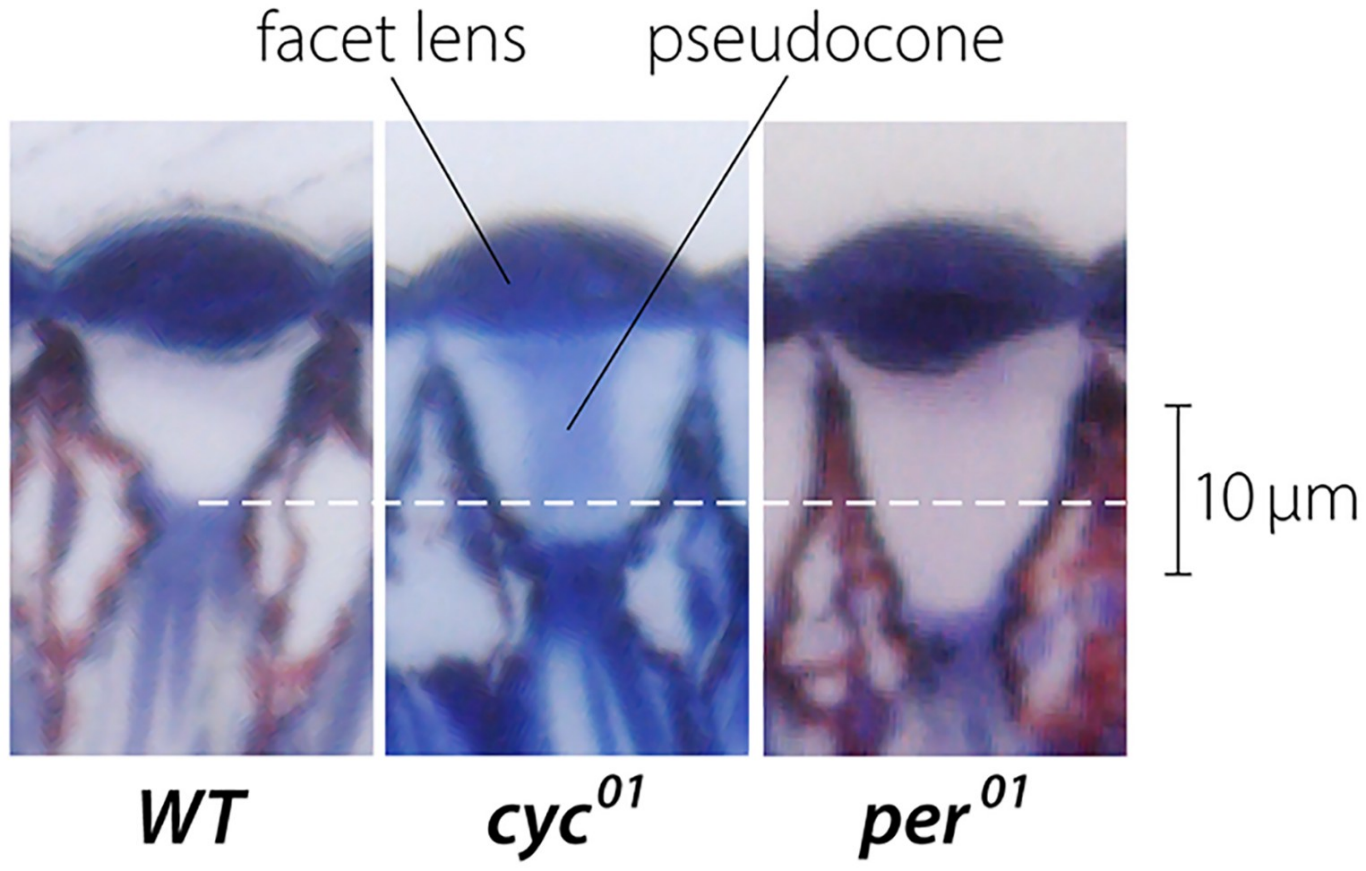
The pseudocones of *Drosophila* ommatidia. Loss of either a positive regulator [*cycle (cyc*^*01*^*)*] or a negative regulator [*period (per*^*01*^*)*] of the clock results in longer pseudocones compared to wild-type (*WT*) flies. Shown here are photomicrographs obtained from 5-day old flies.

To assess the circadian clock in ommatidium development, we studied *Drosophila* mutants lacking either a positive regulator of the clock, *cycle (cyc*^*01*^*)*, or a negative regulator, *period (per*^*01*^*)* [39–41]. At 5 or 20 days of age, the pseudocones of either *cyc*^*01*^ or *per*^*01*^ flies were longer than those of control wildtype flies by 10–27%, depending on genotype and age (Fig 3C, S4 and S5 Tables). Facet lens alterations were less pronounced (Fig 3D–3F, S4 and S5 Tables). At 5 days of age, there were no effects on thickness or diameter of facet lenses comparing either mutant to the control flies; however, the calculated radius of curvature at the anterior facet lens pole was longer in the mutants, an effect reaching statistical significance for the *cyc*^*01*^ flies. The curvature effects were no longer evident at the older age. At 20 days compared to the controls, facet lenses measured 4.6% wider in *per*^*01*^ flies; but their widening was less pronounced and not statistically significant in *cyc*^*01*^ flies. Fig 4 shows longer pseudocones in the mutant flies, compared to wild-type flies.

## Discussion

Disrupting the circadian clock thus elongates optical components of the eye in two phylogenetically distant species, the mouse and the fly, suggesting that at least some component of the mechanism regulating optical development is conserved from flies to mammals. Importantly, these findings support the hypothesis that endogenous circadian rhythms influence signaling pathways that regulate the optical development of the eye [33].

We find that the retinal-specific knockout of the clock gene *Bmal1* induces relative myopia in mice, elongating both the overall axial length and the vitreous cavity of the eye, compared to the eyes of the *Bmal1*^*fl/fl*^ control mice. Similarly, the eyes of *rBmal1* KO mice demonstrate a relative myopia and elongated vitreous chamber compared to the *Chx10*^*cre*^ mice (S2 File S6 and S7 Tables). The negative myopic shift in refraction, longer axial lengths and deeper vitreous chambers of the *rBmal1* KO mouse eyes are the characteristics of much human myopia [47].

In camera-type eyes, refraction changes may result from either a) altered axial dimensions that move the retina relative to the focal position of distant images or b) altered optical powers of the cornea and/or lens that move image position relative to the retina [47]. Refractive errors (e.g., myopia) most commonly follow changes in the axial dimensions of the eye [47]; and experimental myopia in mice frequently manifests a negative shift in refraction values compared to controls (i.e., a relative myopia) [48]. In mice, the anatomical changes in experimental myopia typically are small, like we observed here; but they are optically significant. Based on optical modeling of the mouse eye [49,50], the approximate 5% increase in the mean lengths of the vitreous chambers alone can largely account for the 4–6 diopter myopic shift in *rBmal1* KO mice relative to either control group. Besides vitreous chamber depth, none of the other measured components of the eye clearly explain the myopia of *rBmal1* KO mice compared to each control group. The other parameters either are not consistently different between *rBmal1* KO mice and the two controls or, when different, are not expected to make meaningful contributions to refractive differences based on optical modeling [49]. Thus, the longer vitreous chambers in *rBmal1* KO mice likely account both qualitatively and quantitatively for the myopic shifts in their refractions.

The refractive and vitreous chamber differences between *rBmal1* KO mice and controls are evident at the earliest measurements and persist throughout. Even though mouse eyes are believed to have low spatial resolving power, they seemingly discriminate visually because mouse eyes appropriately adjust to defocus from spectacle lens wear during refractive development [48]. Here, the stable relatively myopic refractions indicate that *rBmal1* KO mice lack the capacity to correct an initial ametropia, even as their vitreous chambers and overall eye lengthens.

The retina in myopic vertebrate eyes exhibits several abnormalities; and recent phenotypings of *rBmal1* KO mice have identified similar retinal changes in mice older than the ones we used here (3–26 months or adult in those studies compared to 10 weeks or less in our study) [35,51]. Neither of these other reports addressed potential refractive anomalies in *rBmal1* KO mice [35,51]. Optical coherence tomography of *rBmal1* KO mice indicates a “slightly reduced” overall retinal thickness (S2 Table and [51]) as noted both in common human myopia [52–55] and also in experimental form-deprivation myopia [56,57]. Loss of *Bmal1* in retina interferes with the spatial pattern, spectral identity and maintenance of cone photoreceptors in mice [58]; indeed, reduced cone density has been measured in *rBmal1* mice at 26 months but not at 3 months of age [51]. Cone density is also reduced in young adult myopic humans [59]. Presumably, these anatomical alterations develop at least in part as a consequence of retinal stretching and thinning to cover the inner surface of the expanded posterior sclera. The findings of thickened cone inner segments and disruption and degeneration of cone outer segment lamellae by 4 weeks of form deprivation myopia in chicks [57] suggests that the disrupted cone photoreceptors in 26 month-old *rBmal1* KOs may be secondary to myopia as well. Stunted dendritic arborizations of rod bipolar cells in 1 month-old *rBmal1* KO mice [51] is another example of altered bipolar cell synapses and/or function in eyes with experimental or clinical myopia [60–62]. Reduced b-waves of scotopic and photopic full-field electroretinograms noted in *rBmal1* KO mice [35,51] likewise occur in myopic humans [63,64]. Although cataracts and corneal abnormalities occur in systemic *Bmal1* KO mice [65,66], *rBmal1* KO mice do not develop these defects in the ocular media [35,51] and experience unimpaired visual input to the retina. *rBmal1* KO mice display normal circadian behavior [35]. The reported small reduction in contrast sensitivity in *rBmal1* KO mice [(51] likely corresponds to “myopic blur”–the central visual problem of all human myopes. The *rBmal1* KO mice studied here were myopic with enlarged vitreous chambers even at the earliest 4-week measurement, and it is possible that many of these reported retinal and visual effects in older mice [35,51] are consequences of the abnormal ocular growth, just as seen in human myopic subjects. Additional investigation of the temporal relation of refraction and these other anomalies, however, will be needed at the early ages to confirm this proposition.

Despite species and model differences, these many parallels strengthen the relevance of *rBmal1* KO mice to other experimental myopia models and, importantly, to myopia in humans. Further, a genome-wide association meta-analysis in humans indicates a role for light-related signaling in the mechanism of refractive errors [67], also consistent with a role for the circadian clock in ametropia.

Similar to the vitreous chamber of the vertebrate camera eye, the pseudocone of the *Drosophila* eye separates the lens facet from the retina, insuring an appropriate focal position for images while exhibiting no intrinsic optical power itself (Fig 3). Others have previously noted this anatomical parallel between camera and appositional eyes [68,69]. Vitreous chamber and pseudocone elongation are the key anatomical features following knockout of the circadian clock in mouse and *Drosophila*, respectively.

The findings in *Drosophila* provide important perspectives for the mouse findings. First, they suggest that the circadian clock that may act independently of vision in optical development. In contrast to camera type vertebrate eyes, pseudocone lengthening is not expected to affect the resolving power of the *Drosophila* eye which depends instead on the inter-ommatidial angle [46,70,71], an anatomical feature not measured here. The optical components of the anterior ommatidia principally concentrate light on the photoreceptors and enhance light sensitivity [72]. A diffraction-dominated optical system, the angular light sensitivity of ommatidia is affected by facet lens diameter; the facet lens diameters here were only 0.67μm larger and only at 20 days in the *per*^*01*^ mutants, an alteration that would reduce the *f*-number by only 4.6% over the ommatidia of the control flies. Given also the waveguide properties of the *Drosophila* rhabdomeres, the axial elongation of the pseudocones likely has minimal effect on the light intensity at the photoreceptor tips [73]. The cone cells and primary pigment cells secrete the lens facet and form the walls of the pseudocone [68,69]. Whether these cells are under circadian control is not known. Nonetheless, from the correspondence of the pseudocone and vitreous chamber (Fig 3A and 3B) [68,69], the *Drosophila* findings suggest a role for the circadian clock in optical development independent of vision.

Underscoring the utility of the *Drosophila* results, *Bmal1* may function as a positive activator of the clock but also more generally as a transcriptional activator, raising the possibility of alternative transcriptional effects. However, pseudocone lengthening in *Drosophila* from knockout of either a positive or a negative clock regulator supports the notion that the phenotypes observed here result from loss of clock function *per se* rather than pleiotropic effects of specific clock genes. Because the circadian clock greatly influences diurnally-regulated functions prominent in the retina [25,74], ascribing the myopia in *rBmal1* KO mice to a clock disorder is consistent with the known role of diurnal rhythms in refractive development and to the alterations in rhythms of ocular dimensions and clock gene expression in experimental models of myopia as discussed in the Introduction [19,22–24].

For some insects, the morphology of the anterior components of ommatidia may undergo a circadian-regulated change between day and night to control the intensity of light at the photoreceptor cells [75,76] or may even differ between diurnal and nocturnal species of insect [77]. To our knowledge, such morphological phenomena have not been reported for *Drosophila* ommatidia and were not investigated here since our flies all were examined in the light adapted state. Nevertheless, investigating the relationship of circadian control of light sensitivity to ocular development may be informative not only because of the available insect data [75,76] but also because of the potential impact of light *per se* and circadian rhythms on mammalian refractive development [12,13,22,33,78].

Relating our *Drosophila* findings to those in mice introduces the notion that appositional invertebrate and camera-type vertebrate eyes may share some mechanisms governing their optical development, at least at a morphologic level. Such parallels, if validated by further investigations, would comprise a remarkable phylogenetic conservation of the developmental processes maintaining images on photoreceptive cells. Thus, genetic manipulation in *Drosophila* could provide a novel system to dissect molecular pathways controlling the optical development of the eye.

Circadian biology could provide a much-needed framework to understand the pathogenesis of myopia [33]. Knowledge of circadian biology is evolving rapidly, and the circadian system regulates the diurnal expression of many genes in a tissue specific pattern [79,80]. Importantly, available data suggest that circadian mechanisms could influence at least some of the pharmacologic and signaling pathways already implicated in myopia [22,33,81]. Repeatedly linked to refractive development [22,38], retinal dopamine is secreted in a diurnal rhythm and entrains intra-retinal signaling to the light:dark cycle [25]. Among the many dopamine receptor subtypes expressed in retina, the D4 dopamine receptor subtype has been found to be under circadian control [82,83]. A non-selective adenosine antagonist, 7-methyl xanthine, is being studied as a potential myopia therapeutic [84]. The retinal expression of the adenosine A2a receptor varies during the day [83], and deletion of the adenosine A2a receptor subtype induces a relative myopia in mice [85]. The GABA (γ-aminobutyric acid) turnover rate, release and specific binding in the retina are all under circadian control [86]; and retinal GABA signaling modulates the amplitude of the retinal circadian rhythms [25]. GABA signaling influences refractive development [87]. The interaction of the cholinergic system and myopia has long been investigated, with most emphasis on the anti-myopia actions of muscarinic antagonist therapies [22,33,81]. More recently, nicotinic acetylcholine receptors also have been implicated in experimental myopia, and passive smoke exposure has been linked clinically to reduced myopia in children [22,33,81]. Circadian mechanisms and cholinergic signaling also interact (reviewed in [88]).

Economically advanced and urbanized societies, where the rising myopia prevalence is of great concern, utilize artificial ambient lighting patterns that weaken and disrupt circadian entraining signals [89,90]. During the day, the indoor environment is comparatively dim, with limited chromaticity compared to outdoors; during the night, artificial lighting both elevates light intensity above natural night and shortens the duration of darkness. Disruptions of circadian entrainment cues are now believed to contribute to a myriad of medical disorders [91–93].

Despite heightened current clinical interest in light exposures and myopia, neither cross-sectional nor prospective clinical investigations of the role of either light or outdoor exposures on refractive development have yet considered the timing of light exposures or their impact on circadian entrainment. Nonetheless, circadian dysregulation may result from many of the environmental factors presumed to provoke myopia, including decreased daytime light exposures from increasing urbanization, less time outdoors and emphasis on schooling; increased ambient light at night from both indoor and outdoor artificial lighting; and either chromatic or other effects from exposure to the screens of electronic devices [94].

The current investigation is based on genetic mutations of the clock, not environmental perturbations. Nevertheless, the results do suggest that the study of circadian disruptions may provide mechanistic links of light to ocular rhythms and refractive development [22,33]. Initial reports even hint toward sleep disturbances in myopic children and adolescents [95–97], behavioral irregularities that may signify an underlying circadian rhythm dysfunction in nearsighted children. These concepts suggest that modern society and its adverse effects on circadian entrainment cues disturb the signaling underlying the ordered optical development of the eye [22,33]. If a circadian dysfunction can be identified in children, it could lead to the introduction of light-based or other behavioral therapies to reduce the incidence or progression of clinical myopia by strengthening circadian rhythms. We thus propose that consideration of circadian biology may provide a novel biological framework to understand myopia pathogenesis, may explain contemporary increases in clinical myopia prevalence, and may provide a means to control clinical myopia with greater efficacy than the approaches now available.

## Supporting information

S1 Table Refractive errors (in diopters) of *Bmal1*^*fl/fl*^ and *rBmal1* KO mice. (DOCX)

S2 Table Ocular dimensions [mean (SEM) in mm] of *Bmal1*^*fl/fl*^ and *rBmal1* KO mice. (DOCX)

S3 Table Corneal radii of curvature (mm) of *Bmal1*^*fl/fl*^ and *rBmal1* KO mice. (DOCX)

S4 Table Anterior ommatidium dimensions (in μm): 5 day-old female *Drosophila*. (DOCX)

S5 Table Anterior ommatidium dimensions (in μm): 20 day-old female *Drosophila*. (DOCX)

S6 Table Refractive errors (in diopters) of Chx10cre and rBmal1 KO mice. (DOCX)

S7 Table Ocular dimensions [mean (SEM) in mm] of Chx10cre and rBmal1 KO mice. (DOCX)

S8 Table Corneal radii of curvature (mm) of Chx10cre and rBmal1 KO mice. (DOCX)

S1 File Supporting materials and methods. (DOCX)

S2 File Refractive development of Chx10cre controls versus rBmal1 KO mice. (DOCX)

S1 Data Mice raw data files. (XLSX)

S2 Data Drosophila raw data files. (XLSX)

## Abbreviations

*Bmal1*: clock gene: brain and muscle arnt-like protein-1; also known as *ARNTL* (aryl hydrocarbon receptor nuclear translocator-like protein 1)
*Bmal1*^*fl/fl*^ mice: mice with exon 8 of the *Bmal1* gene flanked by two loxP sites, littermates of the *rBmal1* KO mice
*Chx10*^*cre*^ mice: mice expressing a single copy of Cre-recombinase driven by the *Chx-10* promoter
*cyc^01^*: mutant *Drosophila* lacking an active *cycle* gene
KO: knock-out
*per^01^*: mutant *Drosophila* lacking an active *period* gene
*rBmal1* KO mice: mice with retinal specific knock-out of *Bmal1*
