# Supplementary material for: Altered ocular parameters from circadian clock gene disruptions
Source: PLoS One. 2019 Jun 18;14(6):e0217111. doi: 10.1371/journal.pone.0217111 (PMC6581257; doi:10.1371/journal.pone.0217111)
Supplement: S1 Table — (DOCX) [file pone.0217111.s001.docx]

| **S1 Table. Refractive Errors (in diopters) of *Bmal1^fl/fl^* and *rBmal1* KO mice** | | |
| --- | --- | --- |
| **Age**  **(weeks)** | ***Bmal1^fl/fl^* (N=10)** | ***rBmal1* KO (N=7)** |
|  | **Mean (SEM)** | **Mean (SEM)** |
| 4 | 3.43 (0.84) | -0.58 (0.54) |
| 6 | 6.87 (1.33) | 0.54 (0.79) |
| 8 | 5.90 (0.64) | 0.77 (0.96) |
| 10 | 4.81 (0.90) | -0.41 (0.79) |
| Repeated measures ANOVA, main effect of genotype: p<0.001.  Data represented in Fig 1A.  N, number of mice. | | |
