# Supplementary material for: Altered ocular parameters from circadian clock gene disruptions
Source: PLoS One. 2019 Jun 18;14(6):e0217111. doi: 10.1371/journal.pone.0217111 (PMC6581257; doi:10.1371/journal.pone.0217111)
Supplement: S2 Table — (DOCX) [file pone.0217111.s002.docx]

| **S2 Table. Ocular Dimensions [mean (SEM) in mm] of *Bmal1^fl/fl^* and *rBmal1* KO mice** | | | | | | | |
| --- | --- | --- | --- | --- | --- | --- | --- |
| **Age (wks)** | **Group** | **Corneal Thickness** | **Anterior Chamber Depth** | **Lens Thickness** | **Vitreous Chamber Depth** | **Retinal Thickness** | **Axial Length** |
|  |  | ANOVA:  Age × genotype interaction: p=n.s | ANOVA:  Age × genotype interaction: p=0.012 | ANOVA:  Main effect of genotype:  p=0.024 | ANOVA:  Main effect of genotype: p<0.001 | ANOVA:  Main effect of genotype: p=0.003 | ANOVA:  Main effect of genotype: p=0.015 |
| 4 | ***Bmal1^fl/fl^*** | 0.075 (0.001) | 0.300 (0.004) | 1.707 (0.006) | 0.655 (0.007) | 0.166 (0.002) | 2.903 (0.015) |
|  | ***rBmal1* KO** | 0.077 (0.002) | 0.300 (0.003) | 1.708 (0.020) | 0.675 (0.014) | 0.151 (0.004) | 2.955 (0.014) |
| 6 | ***Bmal1^fl/fl^*** | 0.082 (0.001) | 0.341 (0.004) | 1.848 (0.008) | 0.633 (0.004) | 0.157 (0.002) | 3.060 (0.013) |
|  | ***rBmal1* KO** | 0.083 (0.003) | 0.330 (0.003)* | 1.875 (0.006) | 0.662 (0.008) | 0.150 (0.004) | 3.102 (0.012) |
| 8 | ***Bmal1^fl/fl^*** | 0.083 (0.001) | 0.363 (0.004) | 1.936 (0.008) | 0.604 (0.004) | 0.160 (0.002) | 3.143 (0.011) |
|  | ***rBmal1* KO** | 0.081 (0.004) | 0.350 (0.003)** | 1.974 (0.008) | 0.635 (0.006) | 0.151 (0.003) | 3.191 (0.014) |
| 10 | ***Bmal1^fl/fl^*** | 0.087 (0.002) | 0.377 (0.002) | 2.000 (0.008) | 0.591 (0.004) | 0.152 (0.003) | 3.207 (0.010) |
|  | ***rBmal1* KO** | 0.084 (0.004) | 0.368 (0.003) | 2.032 (0.013) | 0.611 (0.003) | 0.149 (0.002) | 3.243 (0.015) |
| Data for axial length, vitreous chamber depth, anterior chamber depth, and lens thickness represented in Fig 1B-E.  Number of mice: 10 *Bmal1^fl/fl^*, 7 *rBmal1* KO mice.  Repeated Measures ANOVA, showing main effect or interaction.  Number of asterisks refer to post-hoc comparisons: *p<0.05, **p<0.01; | | | | | | | |
