# Supplementary material for: Altered ocular parameters from circadian clock gene disruptions
Source: PLoS One. 2019 Jun 18;14(6):e0217111. doi: 10.1371/journal.pone.0217111 (PMC6581257; doi:10.1371/journal.pone.0217111)
Supplement: S3 Table — (DOCX) [file pone.0217111.s003.docx]

| **S3 Table. Corneal Radii of Curvature (mm) of *Bmal1^fl/fl^* and *rBmal1* KO mice** | | |
| --- | --- | --- |
| **Age (weeks)** | ***Bmal1^fl/fl^* (N=10)** | ***rBmal1* KO (N=7)** |
|  | **Mean (SEM)** | **Mean (SEM)** |
| 4 | 1.333 (0.008) | 1.349 (0.014) |
| 6 | 1.394 (0.018) | 1.409 (0.010) |
| 8 | 1.447 (0.012) | 1.464 (0.009) |
| 10 | 1.450 (0.008) | 1.494 (0.022) |
| Repeated Measures ANOVA, genotype or interaction effect: p, n.s.  Data represented in Fig 1F.  N, number of mice. | | |
