# Supplementary material for: Altered ocular parameters from circadian clock gene disruptions
Source: PLoS One. 2019 Jun 18;14(6):e0217111. doi: 10.1371/journal.pone.0217111 (PMC6581257; doi:10.1371/journal.pone.0217111)
Supplement: S4 Table — (DOCX) [file pone.0217111.s004.docx]

| **S4 Table. Anterior Ommatidium Dimensions (in µm): 5 Day-Old Female *Drosophila*** | | | | | | | |
| --- | --- | --- | --- | --- | --- | --- | --- |
| **Parameter** | **Wild Type Controls**  **(N=20)** | | ***cyc^01^***  **(N=19)** | | ***per^01^***  **(N=10)** | | **p-value** |
|  | **Mean (SEM)** | **n** | **Mean (SEM)** | **n** | **Mean (SEM)** | **n** |  |
| Pseudocone Length | 9.24 (0.29) | 60 | 11.69 (0.31) | 57 | 11.63 (0.34) | 30 | <0.001 |
| Facet Lens Diameter | 15.15 (0.09) | 60 | 15.39 (0.12) | 57 | 15.48 (0.16) | 30 | 0.25 |
| Facet Lens Thickness | 6.89 (0.07) | 60 | 6.63 (0.08) | 57 | 6.86 (0.11) | 30 | 0.14 |
| Facet Lens Curvature | 16.90 (0.22) | 60 | 18.12 (0.24) | 57 | 17.68 (0.24) | 30 | 0.016 |
| **Post-Hoc Comparisons** | **Comparison Groups** | | | | **Mean Difference**  **(95% CI)** | | **p-value** |
| Pseudocone Length | Wild Type | | *cyc^01^* | | -2.45 (-3.60, -1.31) | | <0.001 |
|  | *cyc^01^* | | *per^01^* | | 0.06 (-1.34, 1.46) | | 0.93 |
|  | Wild Type | | *per^01^* | | -2.39 (-3.78, -1.01) | | 0.001 |
| Facet Lens Diameter | Wild Type | | *cyc^01^* | | -0.24 (-0.61, 0.12) | | 0.19 |
|  | *cyc^01^* | | *per^01^* | | -0.09 (-0.54, 0.36) | | 0.69 |
|  | Wild Type | | *per^01^* | | -0.33 (-0.78, 0.11) | | 0.14 |
| Facet Lens Thickness | Wild Type | | *cyc^01^* | | 0.26 (-0.01, 0.54) | | 0.06 |
|  | *cyc^01^* | | *per^01^* | | -0.23 (-0.57, 0.10) | | 0.17 |
|  | Wild Type | | *per^01^* | | 0.03 (-0.30, 0.36) | | 0.85 |
| Facet Lens Curvature | Wild Type | | *cyc^01^* | | -1.22 (-2.05, -0.39) | | 0.001 |
|  | *cyc^01^* | | *per^01^* | | 0.44 (-0.58, 1.45) | | 0.40 |
|  | Wild Type | | *per^01^* | | -0.78 (-1.78, 0.22) | | 0.13 |
| Data represented in Fig 3C-F.  N, number of flies; n, total number of ommatidium regions measured. To account for replicate measurements within eyes, we conducted overall and post-hoc statistical comparisons using mixed models. See Materials and Methods. | | | | | | | |
