# Supplementary material for: Altered ocular parameters from circadian clock gene disruptions
Source: PLoS One. 2019 Jun 18;14(6):e0217111. doi: 10.1371/journal.pone.0217111 (PMC6581257; doi:10.1371/journal.pone.0217111)
Supplement: S5 Table — (DOCX) [file pone.0217111.s005.docx]

| **S5 Table. Anterior Ommatidium Dimensions (in µm): 20 Day-Old Female *Drosophila*** | | | | | | | |
| --- | --- | --- | --- | --- | --- | --- | --- |
| **Parameter** | **Wild Type**  **Controls**  **(N=10)** | | ***cyc^01^***  **(N=10)** | | ***per^01^***  **(N=9)** | | **p-value** |
|  | **Mean (SEM)** | **n** | **Mean (SEM)** | **n** | **Mean (SEM)** | **n** |  |
| Pseudocone Length | 10.67 (0.35) | 30 | 12.51 (0.37) | 30 | 11.71 (0.26) | 27 | 0.006 |
| Facet Lens Diameter | 14.69 (0.17) | 30 | 15.03 (0.12) | 30 | 15.36 (0.14) | 27 | 0.03 |
| Facet Lens Thickness | 6.19 (0.11) | 30 | 6.36 (0.09) | 30 | 6.61 (0.09) | 27 | 0.13 |
| Facet Lens Curvature | 17.88 (0.29) | 30 | 18.06 (0.28) | 30 | 18.09 (0.25) | 27 | 0.87 |
| **Post-Hoc Comparisons** | **Comparison Groups** | | | | **Mean Difference**  **(95% CI)** | | **p-value** |
| Pseudocone Length | Wild Type | | *cyc^01^* | | -1.87 (-2.99, -0.75) | | 0.002 |
|  | *cyc^01^* | | *per^01^* | | 0.83 (-0.33, 1.98) | | 0.15 |
|  | Wild Type | | *per^01^* | | -1.05 (-2.20, 0.10) | | 0.07 |
| Facet Lens Diameter | Wild Type | | *cyc^01^* | | -0.35 (-0.82, 0.13) | | 0.15 |
|  | *cyc^01^* | | *per^01^* | | -0.33 (-0.81, 0.16) | | 0.19 |
|  | Wild Type | | *per^01^* | | -0.67 (-1.16, -0.18) | | 0.01 |
| Facet Lens Thickness | Wild Type | | *cyc^01^* | | -0.17 (-0.57, 0.23) | | 0.40 |
|  | *cyc^01^* | | *per^01^* | | -0.25 (-0.67, 0.16) | | 0.23 |
|  | Wild Type | | *per^01^* | | -0.42 (-0.84, -0.01) | | 0.046 |
| Facet Lens Curvature | Wild Type | | *cyc^01^* | | -0.18 (-1.03, 0.67) | | 0.68 |
|  | *cyc^01^* | | *per^01^* | | -0.03 (-0.91, 0.84) | | 0.94 |
|  | Wild Type | | *per^01^* | | -0.21 (-1.09, 0.66) | | 0.63 |
| Data represented in Figure 3C-F.  N, number of flies; n, total number of ommatidium regions measured. To account for replicate measurements within eyes, we conducted overall and post-hoc statistical comparisons using mixed models. See Materials and Methods. | | | | | | | |
