# Supplementary material for: Altered ocular parameters from circadian clock gene disruptions
Source: PLoS One. 2019 Jun 18;14(6):e0217111. doi: 10.1371/journal.pone.0217111 (PMC6581257; doi:10.1371/journal.pone.0217111)
Supplement: S6 Table — (DOCX) [file pone.0217111.s006.docx]

| **S6 Table. Refractive Errors (in diopters) of *Chx10^cre^* and *rBmal1* KO mice** | | |
| --- | --- | --- |
| **Age**  **(weeks)** | ***Chx10^cre^* (N=13)** | ***rBmal1* KO (N=7)** |
|  | **Mean (SEM)** | **Mean (SEM)** |
| 4 | 2.98 (0.48) | -0.58 (0.54)** |
| 6 | 2.76 (0.29) | 0.54 (0.79)** |
| 8 | 3.45 (1.02) | 0.77 (0.96)*** |
| 10 | 5.81 (0.57) | -0.41 (0.79)*** |
| Repeated measures ANOVA, interaction effect: p=0.003; main effect of genotype, p=0.003.  Number of symbols indicates significance: **p<0.01, ***p<0.001  N, number of mice. | | |
