# Supplementary material for: Altered ocular parameters from circadian clock gene disruptions
Source: PLoS One. 2019 Jun 18;14(6):e0217111. doi: 10.1371/journal.pone.0217111 (PMC6581257; doi:10.1371/journal.pone.0217111)
Supplement: S8 Table — (DOCX) [file pone.0217111.s008.docx]

| **S8 Table. Corneal Radii of Curvature (mm) of *Chx10^cre^* and *rBmal1* KO mice** | | |
| --- | --- | --- |
| **Age (weeks)** | ***Chx10^cre^* (N=13)** | ***rBmal1* KO (N=7)** |
|  | **Mean (SEM)** | **Mean (SEM)** |
| 4 | 1.416 (0.012)* | 1.349 (0.014) |
| 6 | 1.436 (0.029) | 1.409 (0.010) |
| 8 | 1.417 (0.034) | 1.464 (0.009) |
| 10 | 1.480 (0.027) | 1.494 (0.022) |
| Repeated Measures ANOVA, interaction effect: p=0.004  N, number of mice.  Number of asterisks refer to post-hoc comparisons: *p<0.05 | | |
