# Supplementary material for: Altered ocular parameters from circadian clock gene disruptions
Source: PLoS One. 2019 Jun 18;14(6):e0217111. doi: 10.1371/journal.pone.0217111 (PMC6581257; doi:10.1371/journal.pone.0217111)
Supplement: S1 File — (DOCX) [file pone.0217111.s009.docx]

**S1 File: Supporting Materials and Methods**

**Ocular examinations of mice**

After pupil dilation with topical 1% tropicamide (Bausch & Lomb, Rochester, NY), the refraction of both eyes was measured initially with the mice awake using an automated photorefractor [1]. Each mouse was then sedated (ketamine 80 mg/kg and xylazine 16 mg/kg, ip) and again refracted [62]. Both awake and sedated refractive errors were measured to confirm head positioning and provide back-up measurements if tear film aberration occurred during anesthesia. If the inter-ocular refractive error difference was greater than 2 diopters (D) at p28, the mouse was excluded from the study. While anesthetized, the radius of curvature of the cornea was measured using a photokeratometer [1].

To measure the axial dimensions from a cross-sectional image of the mouse eye, we used a spectral-domain optical coherence tomography (OCT) system (Envisu R4300, Bioptigen, Durham, NC; intrasubject variability: 4±2 µm) calibrated with an overall refractive index of 1.433 [2]. Saline drops were applied to maintain the tear film. A custom Matlab program (MathWorks, Natick, MA) was used to place calipers manually at the interface of each ocular structure. Corneal thickness, lens thickness, and retinal thickness were calculated automatically as the distance between the anterior and posterior surfaces of the cornea, lens, and retina, respectively; the posterior retinal surface is the interface between the retinal pigment epithelium and the choroid. The anterior chamber depth consisted of the distance between the posterior corneal and anterior lens surfaces, and the vitreous chamber depth was the distance between the posterior lens and anterior retinal surfaces. Axial length was calculated as the distance between the anterior corneal surface and the posterior retinal surface. After all ocular measurements were recorded, mice were injected with yohimbine (2.1 mg/kg) to reverse the anesthetizing effects of xylazine; and mice were allowed to recover on a heated pad.

**Preparation and analysis of *Drosophila* ommatidia**

**Preparation** **of ommatidia**

For each *Drosophila* genotype, a culture was started from 10 pairs of parents in vials placed and maintained in an incubator at 25°C and under a 12hr light:12hr dark cycle (500 lux, cool white fluorescent light). When the progeny emerged, 15 pairs of male and female flies were placed in vials and under the same light:dark cycle. Female flies were collected on day 5 and day 20 for processing and analysis.

Following immobilization of the flies on ice, the heads were separated and placed in a fixative solution of 2.5% glutaraldehyde, 2% paraformaldehyde and 0.1M sodium cacodylate buffer, pH 7.4, on ice and then transferred to 4⁰C overnight. The following day the heads were transferred to a fresh fixative solution supplemented with 2% osmium tetroxide and put on ice for 1 hour and then placed in a 2% osmium tetroxide solution for 1 hour on ice. The samples were dehydrated in ethanol, washed with propylene oxide, and then embedded in Durcupan (Sigma-Aldrich, St. Louis, MO) [3]. Horizontal tissue sections were cut through the eyes at 1 micron thickness, collected on glass slides and stained with 1% methylene blue in 1% sodium borate. Digital images were obtained with a photomicroscope (Nikon Microphot SA) at 40X power.

**Analysis of ommatidia**From the photomicrographs, one eye from 10 flies in each cohort was chosen for analysis. Selecting central tissue sections through ommatidia visualized in approximate full length, the dimensions of the facet lenses and pseudocones were measured. The anterior, central and posterior regions of each horizontal section of each eye were analyzed separately, using 6-10 ommatidia/region/fly.

Using Fiji software [42], individual facet lenses were outlined and modeled as an ellipse. The diameter of the facet lenses (defined as the dimension perpendicular to the optic axis) and their thickness (defined as the dimension parallel to the optic axis) were estimated as the major and minor axes of the best-fit ellipses, respectively. An estimate for the radius of curvature at the anterior vertex of the best-fit ellipse was calculated [43] using the relation: curvature = [(major axis/2)^2^]÷(minor axis/2). From the same ommatidia, the lengths of the pseudocones were estimated from a line extending from the posterior-most edge of the facet lens to the deepest location of the pseudocone. Measurements were exported to Excel.

**Supporting Information References**

1. Schaeffel F. Test systems for measuring ocular parameters and visual function in mice. Front Biosci 2008;13:4904-11.

2. Schmucker C, Schaeffel F. In vivo biometry in the mouse eye with low coherence interferometry. Vision Res. 2004;44:2445-56.

3. Gaengel K, Mlodzik M. Microscopic analysis of the adult *Drosophila* retina using semithin plastic sections. Methods Mol Biol. 2008;420:277-87.
