# Supplementary material for: Altered ocular parameters from circadian clock gene disruptions
Source: PLoS One. 2019 Jun 18;14(6):e0217111. doi: 10.1371/journal.pone.0217111 (PMC6581257; doi:10.1371/journal.pone.0217111)
Supplement: S2 File — (DOCX) [file pone.0217111.s010.docx]

**Supporting Information Results**

**Refractive development of *Chx10^cre^* controls *versus rBmal1* KO mice**

The refractions of *rBmal1* KO mice were more myopic than the control *Chx10^cre^* mice at all ages (S6 Table, p=0.003) by an average of 3.57±1.27 diopters. During the study, the refractions of *Chx10^cre^* mice became increasingly hyperopic, from +3.0 to +5.8 diopters. At the end of the study, the *rBmal1* KO mice refractions averaged 6.2 diopters more myopia than *Chx10^cre^* mice (p<0.001) with a gene vs. eye interaction (S6 Table, p=0.003). Consistent with their myopic refractions, the eyes of *rBmal1* KO mice had significantly longer vitreous chamber depths than those of the control *Chx10^cre^* mice (S7 Table, p<0.001), averaging of 0.042±0.009 mm longer over the ages measured. The lens thickness and the axial length each showed an age-by-genotype interaction in comparing *rBmal1* KO and control *Chx10^cre^* mice (S7 Table, p<0.001). Both lens thickness and axial length were greater in *Chx10^cre^* than in *rBmal1* KO mice at the 4-week measurement; but both parameters increased at a faster rate in *rBmal1* KO mice so that the measurements of each parameter were equivalent between the genotypes at the end of the study (S7 Table). The anterior chambers were deeper in the control *Chx10^cre^* mice than in the *rBmal1* KO mice at all times during the study (S7 Table, p=0.016). The corneal radius of curvature showed an age-by-genotype interaction (S8 Table, p=0.004). The cornea was flatter in control *Chx10^cre^* mice than in *rBmal1* KO mice at 4-weeks (S8 Table, post-hoc comparison p<0.05), but their curvatures were comparable at the later measurements. As reviewed in the Discussion, the lengthened vitreous chamber is the ocular component that accounts for the persistent myopia of the *rBmal1* KO mice in comparison to both the *Chx10^cre^* and the *Bmal1^fl/fl^* controls.
